# A Green Treatment Mitigates the Limitations of Coffee Silver Skin as a Filler for PLA/PBSA Compatibilized Biocomposites

**DOI:** 10.3390/molecules29010226

**Published:** 2023-12-31

**Authors:** Davide Perin, Andrea Dorigato, Erica Bertoldi, Luca Fambri, Giulia Fredi

**Affiliations:** Department of Industrial Engineering and INSTM Research Unit, University of Trento, Via Sommarive 9, 38123 Trento, Italy; davide.perin-1@unitn.it (D.P.); andrea.dorigato@unitn.it (A.D.); erica.bertoldi@studenti.unitn.it (E.B.); luca.fambri@unitn.it (L.F.)

**Keywords:** biodegradable composites, poly(lactic acid), poly(butylene succinate-co-adipate), coffee silver skin, filler–matrix interaction

## Abstract

The development of fully renewable and biodegradable composites for short-term applications was pursued by combining a compatibilized poly(lactic acid) (PLA)/poly(butylene succinate-co-adipate) (PBSA) (60:40 wt:wt) blend with coffee silver skin (CSS), an industrial byproduct from coffee processing. An epoxy-based reactive agent (Joncryl ADR-4468) was added as a compatibilizer. CSS was incorporated at 5, 10, and 20 wt% in the blend both in the as-received state and after a simple thermal treatment in boiling water, which was performed to mitigate the negative impact of this filler on the rheological and mechanical properties of the blend. The CSS treatment effectively increased the filler degradation temperature of 30–40 °C, enabling stable melt processing of the composites. It also improved filler–matrix adhesion, resulting in enhanced impact properties (up to +172% increase in impact energy compared to the untreated filler). Therefore, treated CSS demonstrated potential as an effective green reinforcement for PLA/PBSA blends for rigid packaging applications. Future works will focus on studying suitable surface modification of CSS to further increase the interfacial interaction and the tensile quasi-static properties, to fully exploit the capabilities of this renewable material toward the development of eco-friendly composites.

## 1. Introduction

The development of sustainable and eco-friendly materials is becoming increasingly important to address environmental concerns related to plastic waste and to comply with stricter regulations. Biodegradable polymers produced from renewable resources, if properly engineered, could represent a valid solution to progressively replace conventional oil-derived plastics for short-term applications such as single-use packaging, agriculture, and disposable items [1,2,3,4]. Among biodegradable polymers, poly(lactic acid) (PLA) has attracted major interest as it offers good mechanical properties, thermal processability, and transparency [5,6]. However, some issues still limit its wider use, such as its inherent brittleness, poor toughness, narrow processing window, and moderate gas barrier performance [7,8,9].

To overcome these limitations, PLA is often blended with ductile conventional or bioderived polymers [10,11,12,13,14,15]. For example, the blend of PLA and poly(butylene succinate-co-adipate) (PBSA) combines the excellent mechanical properties and processability of PLA with the improved toughness, flexibility, and tear resistance provided by PBSA [16,17,18,19,20,21,22]. However, PLA and PBSA are inherently immiscible due to differences in polarity and crystallization behavior. This results in coarse phase-separated morphologies with poor interfacial adhesion [23]. To improve compatibilization, reactive agents containing epoxy or other reactive moieties can be added to the PLA/PBSA blend to favor the formation of block or graft copolymers at the interface during melt processing [24,25,26,27,28]. For example, Aliotta et al. have recently explored the properties of PLA/PBSA matrices. It was found that the PLA/PBSA blend with 40 wt% PBSA showed the best mechanical performance, with high elongation at break, tear resistance, and impact strength, due to the effective stress transfer between phases provided by the co-continuous morphology. Moreover, adding a commercial epoxy oligomer compatibilizer further enhanced the adhesion and compatibility between the PLA and PBSA phases [17,18].

An attractive option to further improve the sustainability and reduce the cost of PLA/PBSA blends could be the partial replacement of the polymer phase with natural fillers, such as cellulose or lignin derivatives, which do not decrease the biodegradation ability of the biopolymers and can be also used to tune the thermomechanical and functional properties, as shown for several biofiller–biopolymer combinations [29,30,31,32,33,34,35,36]. Among these natural fillers, one that has gained increasing attention in the last years is coffee silver skin (CSS). CSS is an abundant industrial byproduct resulting from coffee bean roasting [37]. It consists primarily of cellulose, hemicelluloses, lignin, and proteins, but it also contains bioactive compounds like chlorogenic acids and peptides with antihypertensive, antioxidant, and antidiabetic properties which may enable uses in nutraceuticals and in the pharma industry [37,38,39,40,41,42].

CSS has also shown potential for incorporation into polymer matrices to decrease cost, increase stiffness, and/or produce active food packaging [43,44]. This concept has been explored especially with biodegradable matrices, such as feather keratin polymer (FKP) [45], poly(3-hydroxybutyrate-co-3-hydroxyvalerate) (PHBV) [46], PHBV/PBAT blends [47,48], and PLA/poly(butylene succinate) (PBS) [49], but also with conventional polymers such as polypropylene (PP) [50]. However, the resulting composites generally report considerable embrittlement, decreased strain at break, and decreased impact properties, resulting especially from the filler agglomeration and the poor filler–matrix interfacial interaction [49]. To the best of the authors’ knowledge, the use of CSS as a filler in PLA/PBSA blends has never been investigated. Moreover, very little has been conducted to improve the interfacial adhesion between CSS and the surrounding polymeric matrix.

This study aims to develop fully biodegradable and eco-sustainable composites by combining a reactively compatibilized PLA/PBSA (60:40) matrix with variable amounts of CSS filler. The first part of the work was devoted to the investigation of the properties of this blend as a function of the amount of a commercial epoxy-based chain extender, i.e., Joncryl ADR-4468 (J) (0, 0.4, and 1 parts per hundred resin, phr). Then, the most promising blend was selected as a matrix for the preparation of the biocomposites at different CSS weight fractions (0, 5, 10, and 20 wt%). Given the negative impact of as-received CSS on the rheological properties of the blend and the poor filler–matrix interaction, a simple thermal treatment in boiling water was applied to CSS to try to mitigate these effects. The results of the characterization of the PLA/PBSA (60:40) blend containing 5, 10, and 20 wt% of treated CSS are also reported in this work.

## 2. Results and Discussion

### 2.1. Characterization of the PLA/PBSA/J Blends

The first part of the work was devoted to investigating the effectiveness of Joncryl (J) as a compatibilizer for the PLA/PBSA (60:40) blend. This was achieved by preparing PLA/PBSA (60:40) blends containing 0, 0.4, or 1 phr J and by subsequently investigating their rheological, microstructural, thermal, and mechanical properties. The list of the prepared compositions is reported in the Materials and Methods section. 

#### 2.1.1. Rheological and Microstructural Properties

**Figure 1:**
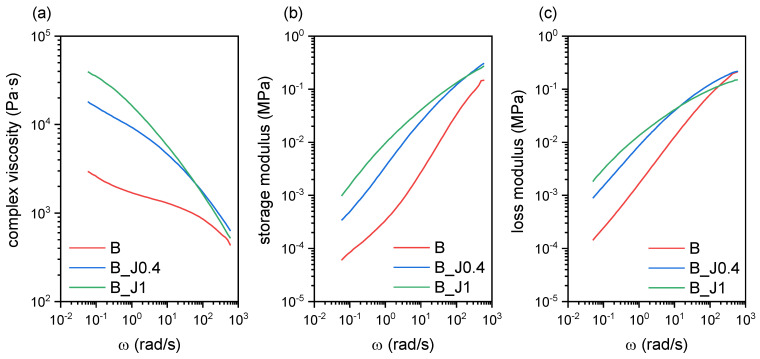
Dynamic rheological behavior of the prepared blends. Trends of (**a**) complex viscosity, (**b**) storage modulus, and (**c**) loss modulus as a function of the angular frequency.

Dynamic rheological tests were carried out to assess the effect of J on the rheological properties of PLA/PBSA blends at a processing temperature of 180 °C. Figure 1 shows the results of the dynamic rheological tests and reports the trends of the complex viscosity $\eta^{*}$, storage modulus (G′), and loss modulus (G″), as a function of the applied frequency for different amounts of J (0 phr, 0.4 phr, and 1 phr).

The PLA/PBSA blend (labeled B, as reported in the Materials and Methods section) shows rheological parameters in line with similar systems reported in the literature [19,20], while the addition of J considerably increases the values of $\eta^{*}$, G′, and G″ throughout the whole investigated frequency range. This implies that J is effective as a compatibilizer/chain extender on the PLA/PBSA blend at the processing temperature investigated in this work. Additionally, the blends containing 0.4 phr and 1 phr J (labeled B_J0.4 and B_J1, respectively) show an increased shear thinning sensitivity, as evidenced by the disappearance of the Newtonian plateau observed for the PLA/PBSA blend. This is also a clear sign of chain extension, enhanced entanglement, and possibly the formation of long-chain branching (LCB), as reported elsewhere for PLA-based systems containing J [13,14,17,51]. This phenomenon is in good agreement with what has been observed during the melt processing, i.e., an increasing torque was appreciated upon the addition of this chain extender.

**Figure 2:**
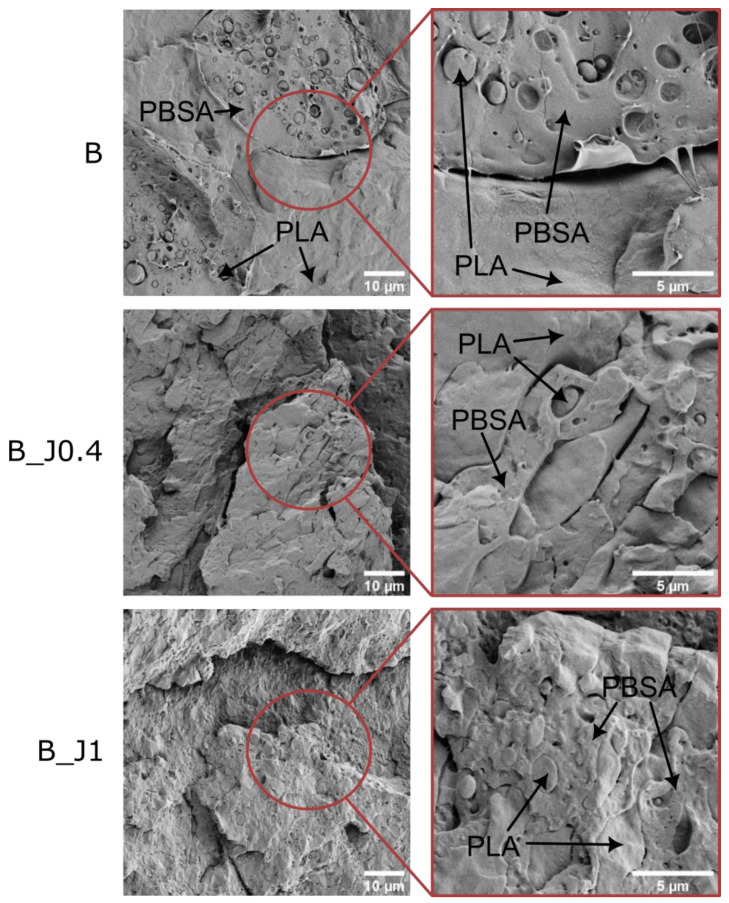
FESEM micrographs of the cryofracture surfaces of the prepared blends at two magnification levels.

Figure 2 shows the SEM micrographs of the prepared blends at two magnification levels. Figure S1 (see Supplementary Materials) shows even lower magnification micrographs. The non-compatibilized blend (sample B) shows a peculiar salami-like structure, with large (>50 μm) PBSA domains including smaller (1–2 μm) PLA islands. This coarse microstructure also shows a non-optimal interfacial adhesion, with evident gaps between the two polymer phases. Remarkable are the microstructural variations when the compatibilizer is added to the blend. Sample B_J0.4 presents a finer microstructure and a higher interfacial adhesion between the PLA and PBSA phases, which demonstrates the effectiveness of J as a compatibilizer for this blend. The microstructural refinement becomes even more apparent at higher J concentrations; sample B_J1 displays a clear co-continuous microstructure, with smaller domain sizes and increased interfacial interaction. The observed variations in microstructure are in line with what has been reported in the literature for similar systems [17,18,22,23,52] and help explain the mechanical results reported in Section 2.1.3.

#### 2.1.2. Thermal Properties

The thermal properties of these blends were investigated with thermogravimetric analysis (TGA) and differential scanning calorimetry (DSC). For brevity’s sake, the results of the TGA tests are reported in the Supplementary Materials (Figure S2; Table S1), while the more interesting DSC results are reported hereafter.

**Figure 3:**
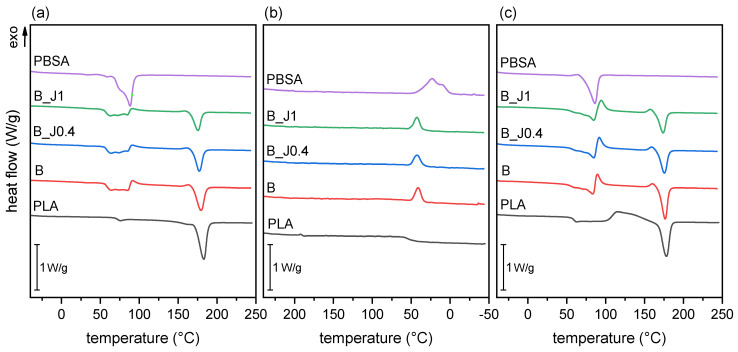
DSC thermograms of neat PLA and PBSA, and the prepared blends (B, B_J0.4, and B_1). (**a**) First heating scan; (**b**) cooling scan; (**c**) second heating scan.

Figure 3 shows the DSC thermograms of the prepared blends (B, B_J0.4, and B_J1), together with those of the neat PLA and PBSA granules, for comparison. The main DSC data are reported in Table 1. Neat PLA and PBSA exhibit typical thermal transitions commonly observed in these polymers [14,17,23,25], except for PLA’s higher *T_g_* (68.4 °C) in the first heating cycle. This is attributed to reversible physical aging phenomena, as demonstrated by the fact that, in the second heating cycle, PLA’s *T_g_* returns to the typical range of 55–56 °C.

The thermograms of the blends are considerably more complex due to the presence of overlapping thermal transitions, but some interesting information can still be drawn. First, PLA’s *T_m_* in the blends is slightly lower than that of neat PLA, and the decrease is more evident with an increase in the J concentration. This convergence of the melting temperatures of the two polymer phases is a sign of compatibilization, as first described by Nishi and Wang [53,54]. Conversely, PLA’s *T_g_* does not seem to be affected by the presence of PBSA or J but only by the aforementioned aging phenomena.

It could be interesting also to determine the impact of blending on the crystallinity of PLA and PBSA. However, in both heating scans, the temperature interval between 55 °C and 120 °C sees PLA’s glass transition, PBSA’s melting, and possibly the PLA’s cold crystallization simultaneously, which complicates the interpretation of the thermograms. More specifically, it is difficult to precisely establish whether and to what extent PLA undergoes cold crystallization in the first heating scan, which makes the precise determination of the PLA’s degree of crystallinity (*χ_c,PLA_*) very difficult. This issue can be partially solved by assuming a linear baseline, which allows for the integration of what comes above the baseline as the PLA’s cold crystallization. This allows for the calculation of the values of *χ_c_* for PLA, which are reported in Table 1. Here, *ΔH_cc,PLA_* is the sum of the cold crystallization peak at approx. 90 °C, partially overlapped with PBSA’s melting, and of the peak just before PLA’s melting, at approx. 165 °C. It can now be observed that, in the produced blends, *χ_c,PLA_* decreases considerably with the concentration of J, from 45.0% of B down to 36.4% of B_J1 (−19%). This is due to the chain extension and branching effect performed by Joncryl on PLA, as reported elsewhere in the literature for similar compatibilized PLA-based systems [13,14]. On the other hand, it is impossible to decouple PBSA’s melting and PLA’s *T_g_* from these DSC scans. This hinders the correct determination of PLA’s *T_g_*, which has still been attempted by measuring the first inflection point. On the other hand, the calculation of PBSA’s *χ_c_* is unfortunately completely prevented.

Nevertheless, the measured variation in the degree of crystallinity of PLA, which is the main polymer phase, is very important as it can affect many mechanical and functional properties such as stiffness, ductility, optical transparency, and gas barrier properties.

#### 2.1.3. Mechanical Properties

**Figure 4:**
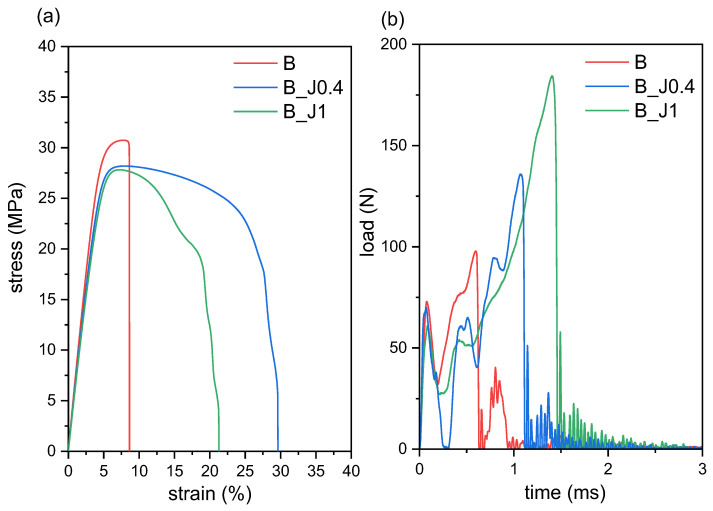
Results of the mechanical tests performed on the prepared blends: (**a**) stress–strain curves obtained from tensile tests; (**b**) load–time curves obtained from Charpy impact tests.

The microstructural refinement and the increased interfacial adhesion evidenced by SEM micrographs, combined with the decreased degree of crystallinity highlighted by DSC, help interpret the results of the mechanical and thermomechanical tests reported in Figure 4a,b and Table 2.

For the tensile tests (Figure 4a), the non-compatibilized blend B exhibits an elastic modulus of 2.0 GPa, a tensile strength of approx. 30 MPa, and a failure immediately after yielding with very limited necking (*ε_b_* = 9%), in line with what has been reported for similar systems [20,26]. The addition of J considerably increases the strain at break (+180% on average) and the post-yield deformation, with only marginal decreases in the material’s stiffness and strength and no significant differences as a function of the J fraction. This proves the compatibilization ability of J on this blend already at 0.4 phr and confirms the conclusions drawn from the microstructural and thermal tests.

The same trends are encountered in the results of the Charpy impact tests (Figure 4b). Adding J considerably increases the maximum impact load and the total energy at break, in line with the quasi-static tensile results and with previous results from the literature on similar systems [17,23]. Unlike the tensile results, though, the effect is significantly higher for a higher J concentration. The trends of *F_max_* and *E_tot,sp_* as a function of the concentration of J are almost linear, and fitting the data with a linear regression gives surprisingly high values of R^2^, i.e., 0.9710 and 0.9987, respectively.

On the other hand, the mechanical behavior of this blend in temperature is negatively impacted by J. As reported in Table 2, the Vicat softening temperature (VST) decreases linearly (R^2^ = 0.9997) with an increasing concentration of J, likely due to the decrease in the crystallinity degree of the PLA phase observed in DSC.

The physical–mechanical characterization of this blend as a function of the J concentration led to the conclusion that J is an effective compatibilizing agent for this blend and, although the microstructural refinement and the blend ductility have margins of improvement with a J content of 1 phr or even higher, the compatibilization can be considered satisfactory already with 0.4 phr J. Such a concentration of J allows the use of the blend B_J0.4 in food-contact applications, in observance of the Food and Drug Administration (FDA) guidelines [55]. Hence, the sample B_J0.4 was used as the matrix for the biodegradable composites produced via the addition of CSS, described in Section 2.2.

### 2.2. Characterization of the PLA/PBSA/J/CSS Composites

As better explained in the Materials and Methods section, the CSS used to prepare the composites has been employed in two different states, i.e., (i) as received and (ii) treated in boiling water for 2 h. The thermal treatment has been performed to counteract the detrimental impact of neat CSS on the rheological behavior and some key mechanical properties of the studied PLA/PBSA blend. Because the most interesting finding of this work is understanding how this treatment influences all the investigated properties, the results of the tests on the composites with untreated and treated CSS will be discussed together, to facilitate the comparison. The list of the prepared compositions is reported in the Materials and Methods section.

#### 2.2.1. Rheological and Microstructural Properties

**Figure 5:**
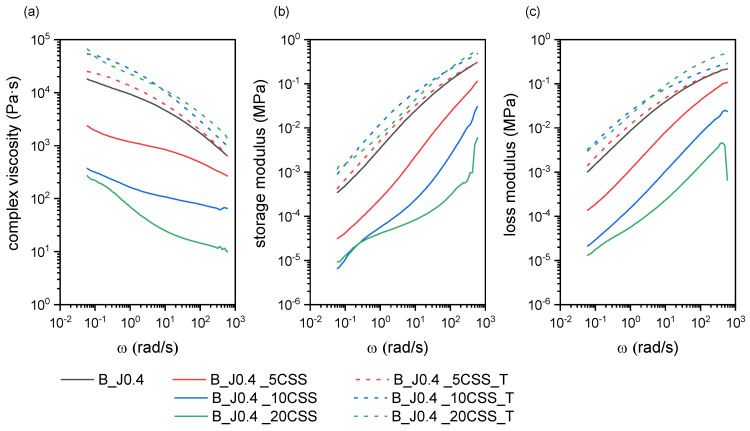
Dynamic rheological behavior of the prepared composites with untreated and treated CSS. Trends of (**a**) complex viscosity, (**b**) storage modulus, and (**c**) loss modulus as a function of the angular frequency.

Figure 5a–c show the results of the dynamic rheological tests for the trends of complex viscosity, storage modulus, and loss modulus for the prepared composites containing untreated or treated CSS. Compared to the neat blend B_J0.4, untreated CSS causes a dramatic decrease in $\eta^{*}$, G′, and G″, and this phenomenon is increasingly evident with an increase in the CSS concentration. This could be the result of a conspicuous degradation of PLA and/or PBSA during melt processing, possibly caused by residual water entrapped in CSS and/or the many organic substances present in the untreated CSS, as thoroughly reported in the literature [38,50]. The degradation of the PLA phase is also evident from the decrease in PLA’s melting temperature, which is observed only for the composites with untreated CSS. The value of *T_m,PLA_* decreases from 176.2 °C of B_J0.4 down to 171.6 °C of B_J0.4_20CSS, while the *T_m,PLA_* measured on the samples of B_J0.4_XCSS_T (X = 5, 10, 20) is on average equal to 177.1 °C (see Supplementary Material, Figure S3 and Table S2).

As will be evident from the discussion of the TGA results (see Section 2.2.2), the treatment performed on CSS does not reduce the water absorption tendency of CSS, but it shifts the thermal degradation onset of this filler to higher temperatures. In other words, While CSS is hygroscopic, the comparable moisture content between untreated and treated CSS rules out water absorption as the key factor. Instead, the untreated CSS likely contains extractable compounds that degrade at the processing temperature of 180 °C, as evidenced by the earlier onset of CSS degradation below 200 °C, as seen in TGA tests, in turn triggering the degradation of the polymer matrix. The extraction in boiling water seems to remove these thermally liable components, thereby expanding the thermal stability window of the treated filler to above the processing temperature. Consequently, composites with treated CSS do not suffer viscosity drops, confirming cleaner blending without polymer degradation. Alternatively, the decrease in complex viscosity may come from the plasticization effect performed by these organic substances on the matrix, even though the concurrent decrease in melting temperature suggests, indeed, degradation. However, because the authors have not found any other works in the literature discussing dynamic rheological tests on PLA-based CSS composites, further investigation is needed to fully understand the reason for this peculiar behavior.

In any case, the result of the addition of treated CSS is an increase in $\eta^{*}$, G′, and G″ with the filler concentration, as expected from a polymer blend filled with solid particles. It is also interesting to notice that this increase in viscosity is not remarkable, which is positive as it does not imply a considerable increase in the pressures and energies involved in a possible industrial process.

**Figure 6:**
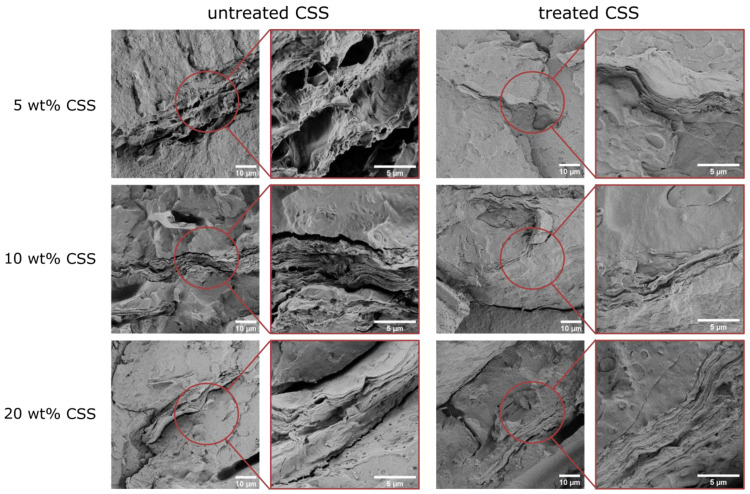
FESEM micrographs of the cryofracture surface of the prepared composites at two different magnifications. Composites containing untreated CSS are shown on the left and those containing treated CSS are shown on the right. The three rows report increasing concentrations of CSS (i.e., 5 wt%, 10 wt%, and 20 wt%).

The treatment of CSS is not only effective in limiting the polymer degradation during melt processing, but it also considerably increases the interfacial interaction with the surrounding matrix. This is clear from the SEM micrographs of the cryofracture surfaces of the prepared composites, displayed in Figure 6. The composites with untreated CSS show poor interfacial adhesion, with evident gaps at the interface, as reported elsewhere in the literature for CSS-containing biopolymers [46,47,48] and other lignocellulosic-filler-reinforced biopolymers [29,32,35]. These interfacial gaps are not present in the composites with treated CSS, which also looks more compact and less porous than the untreated filler. This is at the basis of the higher mechanical performance in Charpy tests, as described in Section 2.2.3.

#### 2.2.2. Thermal Properties

**Figure 7:**
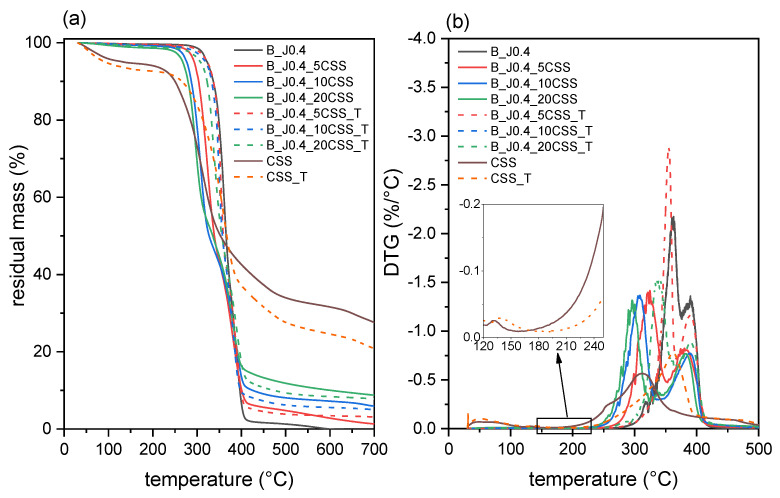
TGA thermograms of the prepared composites: (**a**) residual mass and (**b**) mass loss derivative as a function of temperature.

Figure 7a,b show the TGA thermograms of the prepared composites, while the most important TGA results are reported in Table 3. The derivative thermogravimetry (DTG) thermogram (Figure 7b) has been reported up to 500 °C to improve clarity and give a better overview of the main degradation peaks.

Untreated CSS shows a thermal degradation behavior very similar to what is reported in the literature [46,50]. The first mass loss step, up to 150 °C, is attributed to moisture evaporation, with a mass loss of approx. 6–7 wt%. The subsequent large degradation event, with a peak temperature at 313 °C, is attributed to the degradation of cellulose and hemicellulose, with the hemicellulose fraction degrading in the range of 200–315 °C and the cellulose component in the range of 315–400 °C. Finally, the less intense high-temperature shoulder is associated with the degradation of lignin and proteins [46,48,49,50].

Interestingly, the thermal treatment performed on this filler considerably increases its resistance to thermal degradation. The *T_onset_* is increased from 264 °C to 291 °C and the *T_D_* from 313 °C to 362 °C. More specifically, from the inset in the DTG thermogram (Figure 7b), it is evident that the untreated CSS begins to degrade below 200 °C, while for CSS-T, the degradation starts well above the selected processing temperature (180 °C), thus avoiding the cascading degradation of the PLA fraction during the melt processing. Moreover, DSC scans (see Figure S3 and Table S2 of the Supplementary Materials) show that the melting temperature of PLA decreases with an increasing CSS weight fraction, but only with untreated CSS. This is in good agreement with the results of the rheological tests (see Section 2.2.1) and proves the effectiveness of this simple treatment in expanding the processing temperature range of this interesting biofiller.

The increased thermal resistance of CSS_T is reflected in the increased degradation temperatures of the prepared composites. If the values of *T_onset_* and *T_D_* for the composites containing untreated CSS are significantly lower than that of the unfilled B_J0.4, those of the composites containing treated CSS are only marginally lower. It is interesting to observe that *T_D,PLA_* seems to be more affected than *T_D,PBSA_*, which could suggest a higher impact of the degrading effect of CSS on the PLA phase.

#### 2.2.3. Mechanical Properties

**Figure 8:**
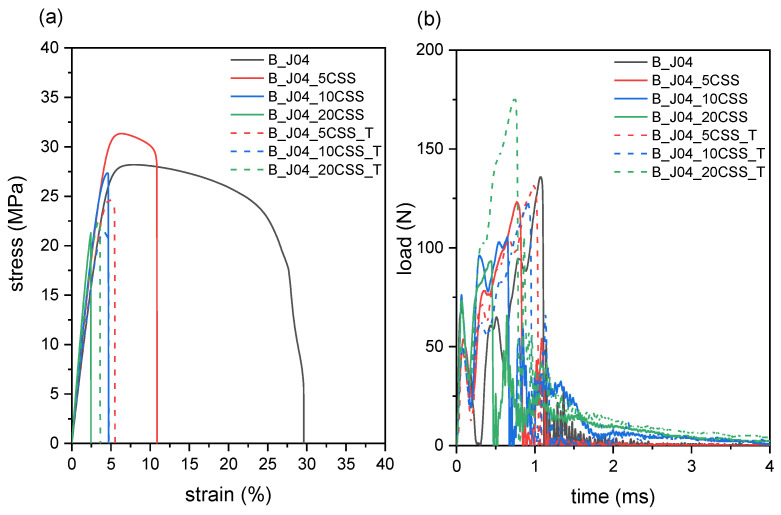
Results of the mechanical tests performed on the prepared composites. (**a**) Stress–strain curves; (**b**) Charpy impact curves with load as a function of time.

Finally, the results of the mechanical tests are shown in Figure 8 and Table 4. The addition of CSS, both untreated and treated, generally promotes a stiffening and an embrittlement of the material, as expected from previous works from the literature dealing with CSS-containing biopolymers [46,49] or other tough polymers combined with lignocellulosic fillers [56,57]. The treatment on CSS does not mitigate this embrittlement, and the composites containing treated CSS show similar or even lower strain at break (*ε_b_*) values than those of the composites with untreated CSS. This effect has been reported elsewhere in the literature for biopolymers reinforced with natural fibers [30]: it has been shown that an increase in the interfacial adhesion between toughened PLA and a rigid fiber may suppress the initiation of toughening mechanisms like the fiber pull out. Even though the filler used in this work cannot be regarded as a fiber due to the very low aspect ratio, this may be an explanation for the slight decrease in all the tensile parameters (elastic modulus *E*, ultimate tensile strength *σ_max_*, and strain at break *ε_b_*) after the filler treatment. The Vicat softening temperature (VST) is also not positively affected by the filler concentration or treatment.

On the other hand, the impact properties do benefit from the CSS treatment. Although the introduction of CSS generally leads to a decrease in *F_max_* and *E_tot,sp_* compared to the unfilled B_J0.4, the CSS treatment helps mitigate this negative effect. Surprisingly, this is especially true at high CSS concentrations. The values of *F_max_* and *E_tot,sp_* for the sample B_J0.4_20CSS_T are comparable to those of the unfilled B_J0.4 and nearly double those of B_J0.4_20CSS. This could be due to the increased, but still not exceptional, interfacial interaction between CSS and the surrounding polymeric matrix, which helps dissipate more energy through crack pinning and crack deflection, as reported for PLA-based systems reinforced with other lignocellulosic fillers [33].

## 3. Materials and Methods

### 3.1. Materials

Two thermoplastic biopolymers were selected for the production of the blend, i.e., poly(lactic acid) (PLA) and poly(butylene succinate-co-butylene adipate (PBSA). PLA Ingeo^®^ 2500 HP was supplied by NatureWorks LLC (Minnetonka, MN, USA) in the form of granules (density = 1.24 g/cm^3^; melt flow rate at 210 °C and 2.16 kg = 8 g/10 min). The selected PBSA was a BioPBS FD92PM, purchased from Mitsubishi Chemical Corporation (Tokyo, Japan). It is a copolymer of succinic acid, adipic acid, and butanediol (density = 1.24 g/cm^3^; melt flow index at 190 °C and 2.16 kg = 4 g/10 min). It is particularly suitable for both blown and cast film extrusion due to its soft and semicrystalline nature. To improve the interaction between the components of the blends, the selected compatibilizer was Joncryl^®^ ADR 4468 (J) (density = 1.08 g/cm^3^ and glass transition temperature = 59 °C). This compatibilizer was purchased by BASF GmbH (Ludwigshafen am Rhein, Germany) in the form of flakes.

The natural filler selected in this work was coffee silver skin (CSS), kindly provided by Cesare Trucillo Spa (Salerno, SA, Italy). Due to its nature as an industrial byproduct, it appeared as compacted blocks of small powder. Thus, it was manually ground and sieved down to a particle size of less than 300 µm. The obtained powder was labeled as CSS. The CSS was employed both as received and after a thermal treatment. The treatment consisted of boiling the powder in water for 2 h under constant magnetic stirring. The obtained powder was filtered, dried, and sieved again to remove agglomerates bigger than 300 µm. This simple and green treatment had the objective of removing oils and possible contaminants.

### 3.2. Sample Preparation

To avoid any hydrolytic degradation during the mixing operations, PLA, PBSA, and J granules were carefully dried in a vacuum oven at 60 °C for 12 h, while CSS powder was dried in a vacuum oven at 80 °C for 12 h. The blends were prepared by adding PLA and PBSA granules at a constant relative ratio (60/40 wt/wt) in a Thermo Haake Rheomix 600 internal mixer equipped with counter-rotating rotors, operating at 60 rpm at a temperature of 180 °C. After one minute, different amounts of J were added to the mixer, and the blend was processed for a total processing time of 10 min. For the blends containing CSS, different amounts of this filler were added to the blend after 5 min. All the obtained blends were subsequently compression molded in a Carver hot plate press at 180 °C for 5 min, under an applied pressure of 3.4 MPa. Through this methodology, two different square sheets were prepared for each composition, having dimensions of 120 × 120 × 2 mm^3^ and 100 × 100 × 4 mm^3^, respectively. Table 5 reports all the prepared compositions and the corresponding nominal weight fractions of each constituent. The compositions were chosen to ensure a constant compatibilizer-to-biopolymer ratio, as the amount of J is always 0.4 g for every 100 g of the total biopolymer fraction (PLA+PBSA), i.e., 0.4 parts per hundred resin (phr).

### 3.3. Characterization

#### 3.3.1. Rheological Properties

Dynamic rheological measurements were conducted using an HR-2 Discovery Hybrid Rheometer (TA Instruments, New Castle, DE, USA) in the parallel plate configuration (diameter of the plates = 25 mm, gap = 2.0 mm). Frequency sweep tests were performed at 180 °C, in air, under a frequency range from 0.05 to 600 rad/s, and 1% of strain amplitude. The trends of storage modulus (*G′*), loss modulus (*G″*), and complex viscosity (η*) were determined as a function of frequency. At least three specimens were tested for each composition.

#### 3.3.2. Microstructural Properties

Field emission scanning electron microscopy (FESEM) micrographs of the cryofractured surfaces of the specimens were acquired using a Zeiss Supra 40 microscope (Carl Zeiss AG, Oberkochen, Germany), with an accelerating voltage of 2.5 kV. Prior to the analysis, a Platinum–Palladium (80:20) conductive coating was sputtered onto the specimens.

#### 3.3.3. Thermal Properties

Thermogravimetric analysis (TGA) was carried out on all the blends and all the constituents using a TG50 MT5 (Metter-Toledo, Columbus, OH, USA) thermobalance, with a nitrogen flow of 100 mL/min and a heating rate of 10 °C/min from 30 °C to 700 °C. The test allowed for the determination of the onset degradation temperature after water evaporation (*T_onset_*); the temperatures corresponding to a mass loss of 1 wt%, 3 wt%, and 5 wt% (*T_1%,_ T_3%_,* and *T_5%_*); the temperature at the maximum degradation kinetics as the peak temperature of the derivative thermogravimetry (DTG) thermogram (*T_D_*); and the residual mass after the test (*m_r,700_*). One specimen was tested for each composition.

Differential scanning calorimetry (DSC) tests were performed using a Mettler DSC30 instrument (Metter-Toledo,Columbus, OH, USA) under a nitrogen flow of 100 mL/min, in a temperature range from −50 to 250 °C, with a heating/cooling rate of 10 °C/min. Each specimen was subjected to a first heating scan, a cooling scan, and a second heating scan. From the obtained thermograms, the thermal transitions of the constituents of the blends were measured, i.e., the glass transition temperature of PLA (*T_g,PLA_*), the melting temperatures and enthalpy of PBSA (*T_m,PBSA_* and *ΔH_m,PBSA_*), the cold crystallization temperature and enthalpy of PLA (T_cc,PLA_ and *ΔH_cc,PLA_*), and the melting temperatures and enthalpy of PLA (*T_m,PLA_* and *ΔH_m,PLA_*). Data in the first heating scan were used to evaluate the degree of crystallinity of PLA (*χ*), following Equation (1):

(1) $$\chi =\frac{{\Delta H}_{m}-{\Delta H}_{cc}}{{\Delta H}_{m}^{*}\;\cdot \;\omega_{PLA}}\;\cdot 100$$

where $\;{\Delta H}_{m}^{*}\;$ is the theoretical melting enthalpy, equal to 93.7 J/g for PLA [58] and *ω_PLA_* is the nominal weight fraction of PLA in the blends (as listed in Table 5). One specimen was tested for each composition.

#### 3.3.4. Mechanical and Thermomechanical Properties

Vicat softening temperature (VST) was determined through an ATS-FAAR mod. MP/3 machine (Milan, Italy). Rectangular samples 20 × 10 × 4 mm^3^ were tested in the temperature range of 30–150 °C, with an applied load of 10 N and a heating rate of 120 °C/min. At least 4 specimens were tested for each composition.

Quasi-static tensile tests were performed using an Instron^®^ 5969 dynamometer (Norwood, MA, USA) equipped with a 1 kN load cell. The standard ISO 527 was used for the quasi-static tests and the test was performed on 1BA specimens laser-cut from the 2 mm thick sheets. The tests were performed at a crosshead speed of 10 mm/min, and at least ten specimens were tested for each composition until breakage. Then, the maximum stress (*σ_max_*) and the strain at break (*ε_b_*) were evaluated. For the determination of the elastic modulus (*E*), quasi-static tensile tests were performed using the same machine equipped with an Instron^®^ 2620-601 extensometer, with a gauge length of 12.5 mm, at a crosshead speed of 0.25 mm/min. As reported in the standard, the elastic modulus was calculated as the secant modulus considering the stress levels associated with the strain values of 0.05% and 0.25%.

Charpy impact tests were performed following the standard ISO 179. The specimens had dimensions of 80 × 10 × 4 mm^3^, with a notch length of 2 mm and a span length of 62 mm. At least ten specimens were tested for each composition. The tests were performed using an instrumented CEAST Charpy impact machine. The tests were performed with an impactor of 1.187 kg, placed at a starting angle of 150°, hitting the specimen at 2.5 m/s. The load–displacement curves allowed for the determination of the maximum load sustained by the specimen (*F_max_*) and the total specific energy (*E_tot,sp_*), obtained by dividing the total absorbed energy by the load-bearing cross-section.

## 4. Conclusions

This work focused on developing fully renewable composites by incorporating treated coffee silver skin (CSS), an industrial byproduct from coffee processing, into PLA/PBSA polymer blends. Initial compatibilization of the 60:40 PLA/PBSA matrix with 0.4 phr of the chain extender Joncryl effectively refined the phase morphology and nearly tripled the blend elongation at break (ε_b_ = 28%) and the absorbed impact energy (E_tot,sp_ = 5.1 kJ/m^2^) while marginally decreasing stiffness and strength. While further increasing Joncryl enhanced the impact properties, it did not significantly increase tensile properties and it progressively deteriorated the heat deflection temperature.

Untreated CSS incorporation severely degraded composite properties by promoting polymer degradation during melt processing, evidenced by a 50–70% viscosity decrease in rheology along with a 60 °C lower thermal degradation onset at 20 wt% CSS loading. Moreover, untreated CSS sharply embrittled the composites, decreasing elongation at break by 85% and impact energy absorption by 65% at 20 wt% CSS. This was probably also due to the poor interfacial adhesion, as indicated by the large gaps at the CSS–matrix interface detected with SEM.

Alternatively, a simple and sustainable boiling water treatment to remove reactive CSS impurities and thermally liable compounds prevented such drastic viscosity drops during compounding and restricted thermal degradation to only 20 °C at 20 wt% CSS content. This allowed for the effective valorization of CSS as a renewable reinforcement. Compared to untreated CSS, the treated filler appeared more compact with enhanced matrix adhesion, doubling the maximum impact load and tripling the absorbed energy at 20 wt% CSS due to improved stress transfer. However, embrittlement effects persisted under tensile loading after treatment.

Overall, with further interface enhancements to retain ductility, the boiling water extraction enables exploiting CSS as filler to develop eco-friendly PLA/PBSA rigid composites, providing valuable insights to tap the potential of this abundant biowaste for renewable food packaging.

## Figures and Tables

**Table 1:** Main results of the DSC tests on neat PLA, neat PBSA, and the prepared blends (B, B_J0.4, and B_1). n.d. = not detectable; n.a. = not applicable.

Sample	First Heating Scan							Cooling Scan		Second Heating Scan				
	*T_g,PLA_* (°C)	*T_m,PLA_* (°C)	*ΔH_m,PLA_* (J/g)	*ΔH_cc,PLA_* (J/g)	*χ_c,PLA_* (%)	*T_m,PBSA_* (°C)	*ΔH_m,PBSA_* (J/g)	*T_c,PBSA_* (°C)	*ΔH_c,PBSA_* (J/g)	*T_g,PLA_* (°C)	*T_m,PLA_* (°C)	*ΔH_m,PLA_* (J/g)	*T_m,PBSA_* (°C)	*ΔH_m,PBSA_* (J/g)
PLA	68.4	181.9	59.1	0	63.0	n.a.	n.a.	n.a.	n.a.	55.9	177.1	48.6	n.a.	n.a.
B	54.8	178.5	32.1	2.8	45.0	n.d.	n.d.	40.6	15.6	55.2	175.2	36.5	n.d.	n.d.
B_J0.4	57.2	176.2	30.0	7.3	40.4	n.d.	n.d.	42.6	15.0	57.1	174.5	27.7	n.d.	n.d.
B_J1	56.6	174.7	23.8	2.5	36.4	n.d.	n.d.	42.3	14.6	56.8	173.0	25.8	n.d.	n.d.
PBSA	n.a.	n.a.	n.a.	n.a.	n.a.	88.8	63.2	23.2	47.2	n.a.	n.a.	n.a.	85.3	46.1

**Table 2:** Main results of the tensile, Charpy, and Vicat tests on the prepared blends.

Sample	Tensile Test			Charpy Test		Vicat Test
	*E* (GPa)	*σ_max_* (MPa)	*ε_b_* (%)	*F_max_* (N)	*E_tot,sp_* (kJ/m^2^)	VST (°C)
B	2.0 ± 0.1	29.6 ± 0.9	9 ± 2	99 ± 13	3.2 ± 0.5	123 ± 8
B_0.4J	1.9 ± 0.1	29.4 ± 1.1	28 ± 16	144 ± 12	5.1 ± 0.8	106 ± 2
B_1J	1.8 ± 0.1	28.6 ± 1.3	23 ± 14	181 ± 7	7.6 ± 0.8	82 ± 4

**Table 3:** Main results of the TGA tests of the prepared composites.

Sample	*T_onset_* (°C)	*T_D,PLA_* (°C)	*T_D,CSS_* (°C)	*T_D,PBSA_* (°C)	*m_r_,_700_* (%)
B_J0.4	341	351	-	379	0.0
B_J0.4_5CSS	300	315	-	369	1.3
B_J0.4_10CSS	284	299	-	379	5.9
B_ J0.4_20CSS	276	287	-	371	8.8
B_ J0.4_5CSS_T	341	355	-	388	3.1
B_J0.4_10CSS_T	338	349	-	383	5.0
B_J0.4_20CSS_T	320	338	-	390	7.8
CSS	264	-	313	-	27.6
CSS_T	291	-	362	-	20.8

**Table 4:** Main results of the tensile, Charpy, and Vicat tests on the prepared composites.

Sample	Tensile Test			Charpy Test		Vicat Test
	E (GPa)	σ_max_ (MPa)	ε_b_ (%)	F_max_ (N)	E_tot,sp_ (kJ/m^2^)	VST (°C)
B_0.4J	1.9 ± 0.1	29.4 ± 1.1	28.0 ± 16.0	144 ± 12	5.1 ± 0.8	105.6 ± 1.8
B_0.4J_5CSS	2.2 ± 0.1	31.4 ± 0.7	12.2 ± 3.9	124 ± 7	3.6 ± 0.7	85.2 ± 7.3
B_0.4J_10CSS	2.4 ± 0.1	27.2 ± 1.1	4.6 ± 0.4	105 ± 6	3.1 ± 0.2	85.8 ± 5.0
B_0.4J_20CSS	3.1 ± 0.1	21.6 ± 0.2	2.4 ± 0.1	85 ± 5	1.8 ± 0.2	95.8 ± 0.7
B_0.4J_5CSS_T	1.9 ± 0.1	25.3 ± 0.5	5.8 ± 1.7	106 ± 17	5.0 ± 0.9	88.6 ± 10.1
B_0.4J_10CSS_T	1.6 ± 0.2	20.8 ± 1.0	4.5 ± 0.2	110 ± 16	5.2 ± 1.0	92.7 ± 8.9
B_0.4J_20CSS_T	2.0 ± 0.1	19.4 ± 3.1	3.2 ± 0.5	144 ± 12	4.9 ± 0.6	94.5 ± 2.0

**Table 5:** List of the prepared compositions with nominal weight fractions of the constituents.

Sample	PLA (wt%)	PBSA (wt%)	J (phr)	CSS (wt%)	State of CSS
B	60	40	0	-	-
B_0.4J	60	40	0.4	-	-
B_1J	60	40	1.0	-	-
B_0.4J_CSS5	57	38	0.4	5	As received
B_0.4J_CSS10	54	36	0.4	10	As received
B_0.4J_CSS20	48	32	0.4	20	As received
B_0.4J_CSS5_T	57	38	0.4	5	Treated
B_0.4J_CSS10_T	54	36	0.4	10	Treated
B_0.4J_CSS20_T	48	32	0.4	20	Treated

phr = parts per hundred resin, i.e., grams of J every 100 g of polymer (PLA+PBSA).

## Data Availability

The data presented in this study are available on request from the corresponding author. The data are not publicly available due to confidentiality issues and the fear of misinterpretation or misuse.

## Supplementary Materials

The following supporting information can be downloaded at https://www.mdpi.com/article/10.3390/molecules29010226/s1, Figure S1: Low-magnification micrographs of the PLA/PBSA/J blends; Figure S2: TGA thermograms of the neat PLA, PBSA, and Joncryl and the prepared blends. (a) Residual mass and (b) mass loss derivative; Figure S3: DSC thermograms (first heating scan) of the matrix B_J0.4 and the prepared CSS-containing composites; Table S1: Results of the TGA tests on the neat PLA, PBSA, and Joncryl and on the prepared blends; Table S2: Main results of the DSC tests (first heating scan) on the matrix B_J0.4 the prepared composites.
