# Supplementary material for: A Green Treatment Mitigates the Limitations of Coffee Silver Skin as a Filler for PLA/PBSA Compatibilized Biocomposites
Source: Molecules. 2023 Dec 31;29(1):226. doi: 10.3390/molecules29010226 (PMC10780561; doi:10.3390/molecules29010226)
Supplement: Supplementary file 1 [file molecules-29-00226-s001.zip › molecules-2749942-supplementary.pdf]

Supplementary materials

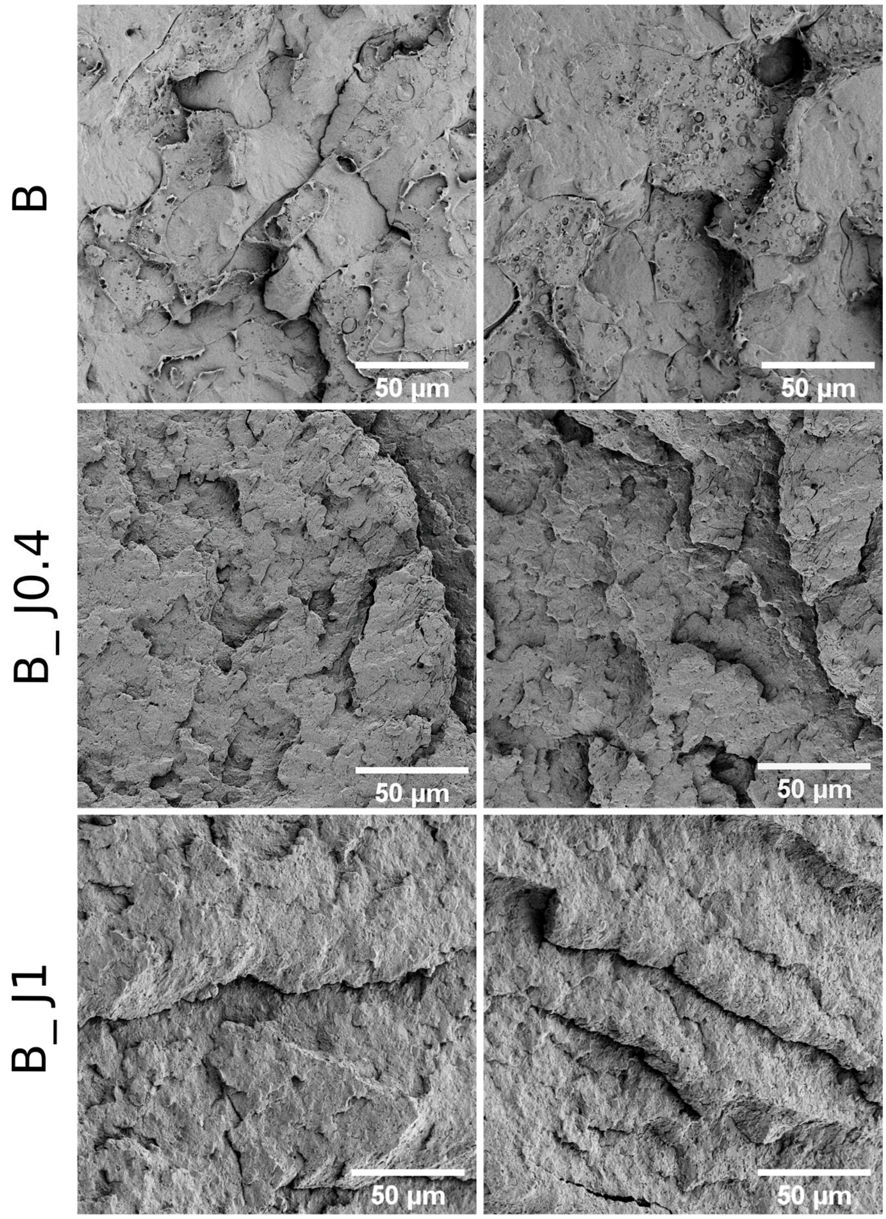

Figure S1. Low magnification SEM of PLA/PBSA/J blends

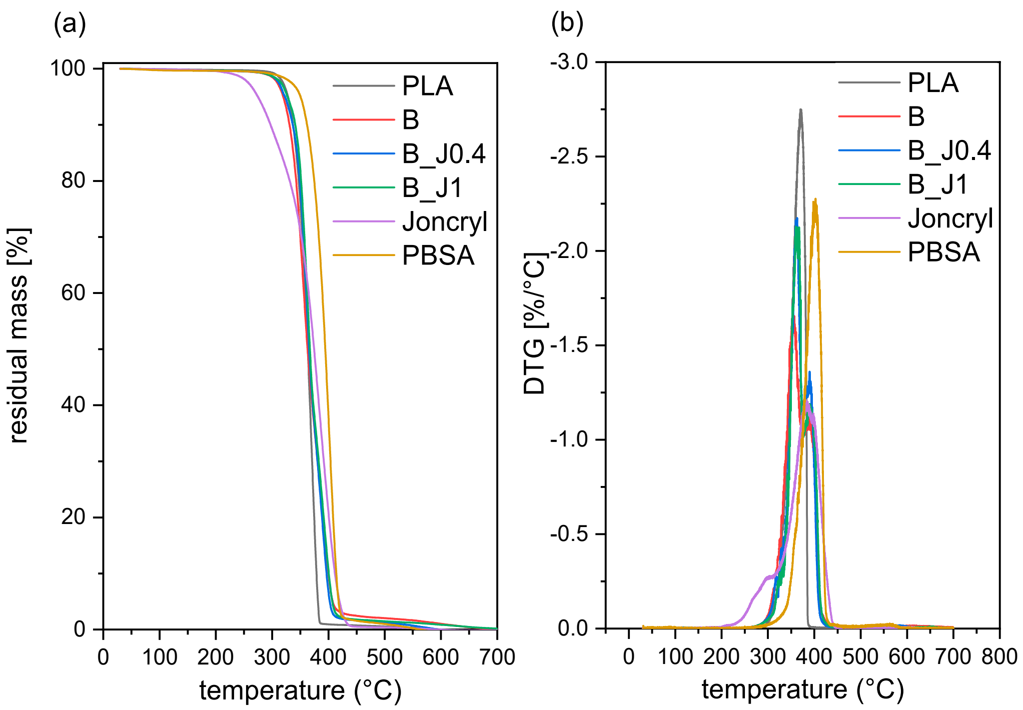

Figure S2. TGA thermograms of the neat PLA, PBSA, and Joncryl and the prepared blends. (a) Residual mass and (b) mass loss derivative

Table S1.: Results of the TGA tests on the neat PLA, PBSA, and Joncryl and on the prepared blends.

**Sample**	$T_{1\%}$ **(°C)**	$T_{3\%}$ **(°C)**	$T_{5\%}$ **(°C)**	$T_{D,PLA}$ **(°C)**	$T_{D,PBSA}$ **(°C)**	$T_{D,Joncryl}$ **(°C)**	$m_{r,700}$ **(%)**
PLA	297	312	318	361	-	-	0.0
B	281	302	310	346	379	-	0.0
B_0.4J	283	305	313	351	379	-	0.0
B_1J	283	309	317	355	380	-	0.0
Joncryl	232	262	275	-	-	382	0.0
PBSA	294	329	340	-	391	-	0.0

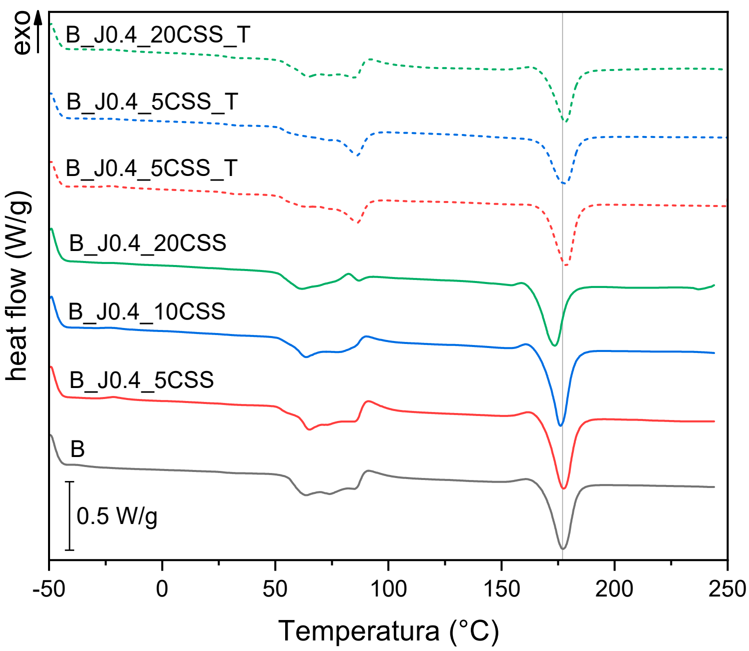

Figure S3. DSC thermograms (first heating scan) of the matrix B_J0.4 and the prepared composites.

Table S2. Main results of the DSC tests (first heating scan) on the prepared composites.

**Sample**	$T_{g,PLA}$ **(°C)**	$T_{m,PLA}$ **(°C)**	$\Delta H_{m,PLA}$ **(J/g)**	$\Delta H_{cc,PLA}$ **(J/g)**	$\chi_{c,PLA}$ **(%)**
B_J0.4	57.2	176.2	30.0	7.3	40.4
B_J0.4_5CSS	52.1	176.0	29.9	5.5	45.7
B_J0.4_10CSS	60.0	174.4	25.1	3.5	42.7
B_J0.4_20CSS	52.8	171.6	25.1	2.5	50.3
B_J0.4_5CSS_T	53.0	177.5	26.5	0	49.6
B_J0.4_10CSS_T	52.9	176.9	23.0	0	45.5
B_J0.4_20CSS_T	50.3	176.7	21.4	3.4	40.0
